# Glucose-dependent and Glucose-sensitizing Insulinotropic Effect of Nateglinide: Comparison to Sulfonylureas and Repaglinide

**DOI:** 10.1155/EDR.2001.63

**Published:** 2001

**Authors:** Shiling Hu, Shuya Wang, Beth E. Dunning

**Affiliations:** 1 Metabolic and Cardiovascular Diseases Novartis Institute for Biomedical Research 556 Morris Avenue Summit NJ 07901 USA

## Abstract

Nateglinide, a novel D-phenylalanine derivative, stimulates insulin release *via* closure of K_ATP_ channels in pancreatic *β*-cell, a primary mechanism of action it shares with sulfonylureas (SUs) and repaglinide. This study investigated (1) the influence of ambient glucose levels on the insulinotropic effects of nateglinide, glyburide and repaglinide, and (2) the influence of the antidiabetic agents on glucose-stimulated insulin secretion (GSIS) *in vitro* from isolated rat islets. The EC_50_ of nateglinide to stimulate insulin secretion was 14 μM in the presence of 3mM glucose and was reduced by 6-fold in 8mM glucose and by 16-fold in 16mM glucose, indicating a glucose-dependent insulinotropic effect. The actions of glyburide and repaglinide failed to demonstrate such a glucose concentration-dependent sensitization. When tested at fixed and equipotent concentrations (~2x EC_50_ in the presence of 8mM glucose) nateglinide and repaglinide shifted the EC_50_s for GSIS to the left by 1.7mM suggesting an enhancement of islet glucose sensitivity, while glimepiride and glyburide caused, respectively, no change and a right shift of the EC_50_. These data demonstrate that despite a common basic mechanism of action, the insulinotropic effects of different agents can be influenced differentially by ambient glucose and can differentially influence the islet responsiveness to glucose. Further, the present findings suggest that nateglinide may exert a more physiologic effect on insulin secretion than comparator agents and thereby have less propensity to elicit hypoglycemia *in vivo*.


*Int. Jnl. Experimental Diab. Res.*, Vol. 2, pp. 63–72
Reprints available directly from the publisher
Photocopying permitted by license only

© 2001 OPA (Overseas Publishers Association) N.V.
Published by license under
the Harwood Academic Publishers imprint,
part of Gordon and Breach Publishing,
a member of the Taylor & Francis Group.
Printed in the United States.

### Glucose-dependent and Glucose-sensitizing Insulinotropic Effect of Nateglinide: Comparison to Sulfonylureas and Repaglinide

SHILING HU*, SHUYA WANG and BETH E. DUNNING

*Metabolic and Cardiovascular Diseases, Novartis Institute for Biomedical Research, Summit, USA*

*(Received 6 December 2001; In final form 18 January 2001)*

**Nateglinide, a novel D-phenylalanine derivative, stimulates insulin release *via* closure of $K_{ATP}$ channels in pancreatic $\beta$-cell, a primary mechanism of action it shares with sulfonylureas (SUs) and repaglinide. This study investigated (1) the influence of ambient glucose levels on the insulinotropic effects of nateglinide, glyburide and repaglinide, and (2) the influence of the antidiabetic agents on glucose-stimulated insulin secretion (GSIS) *in vitro* from isolated rat islets. The $EC_{50}$ of nateglinide to stimulate insulin secretion was 14 μM in the presence of 3 mM glucose and was reduced by 6-fold in 8 mM glucose and by 16-fold in 16 mM glucose, indicating a glucose-dependent insulinotropic effect. The actions of glyburide and repaglinide failed to demonstrate such a glucose concentration-dependent sensitization. When tested at fixed and equipotent concentrations (~2x $EC_{50}$ in the presence of 8 mM glucose) nateglinide and repaglinide shifted the $EC_{50}$s for GSIS to the left by 1.7 mM suggesting an enhancement of islet glucose sensitivity, while glimepiride and glyburide caused, respectively, no change and a right shift of the $EC_{50}$. These data demonstrate that despite a common basic mechanism of action, the insulinotropic effects of different agents can be influenced differentially by ambient glucose and can differentially influence the islet responsiveness to glucose. Further, the present findings suggest that nateglinide may exert a more physiologic effect on insulin secretion than comparator agents and thereby have less propensity to elicit hypoglycemia *in vivo*.**

*Keywords*: Rat pancreatic islets; Nateglinide; Glucose stimulated insulin secretion; Glucose sensitivity; Static incubation

### INTRODUCTION

The homeostatic maintenance of blood glucose concentration is an integrated process predominantly regulated by the anti-hyperglycemic hormone insulin. When blood glucose rises, uptake of glucose into the $\beta$-cells leads to an elevation of ATP/ADP ratio and a sequence of ionic events. The resultant increase in intracellular $Ca^{2+}$ triggers exocytosis and insulin release.[1] The generation of insulin occurs through a precursor, proinsulin, whose biosynthesis is stimulated by nutrient secretagogue like glucose.[2] Aside from nutrient secretagogues, there are a number of non-nutrient

*Address for correspondence: Metabolic and Cardiovascular Diseases, Novartis Institute for Biomedical Research, 556 Morris Avenue Summit, NJ 07901, USA. Tel.: 908-277-5703, Fax: 908-277-4756, e-mail: shiling.hu@pharma.novartis .com agents, which exert insulinotropic action *via* mechanisms other than stimulating the biosynthesis of insulin. As representatives of a class of non-nutrient insulin secretagogues, sulfonylureas (SUs) like glyburide (GLY) and glimepiride (GLI) act on pancreatic $\beta$-cells by blocking $K_{ATP}$ channels.[3–5] Agents that share the primary mechanism of action with SUs include repaglinide (REP), a non-SUs benzoic acid derivative,[6–8] and nateglinide (NAT), a novel oral hypoglycemic agent recently marketed.[9,10] The structures of these hypoglycemic agents are shown in Figure 1.

In treating type 2 diabetes, SUs and REP can cause long-lasting hypoglycemia under both normoglycemic and hyperglycemic conditions in animal models.[11,12] NAT, on the other hand, demonstrates an enhanced activity under hyperglycemic conditions due to glucose-sensitive action.[9,13,14] In line with the *in vivo* data, our earlier study characterizing the $K_{ATP}$ channel-blocking effect by hypoglycemic drugs showed that NAT but not GLY and REP had an increased potency at elevated glucose concentration.[15] The aim of the present study was to obtain further evidence for a glucose-sensitive insulinotropic action by NAT. We investigated the interaction between glucose and NAT with regard to the stimulation of insulin release *in vitro* from rat pancreatic islets by determining the influence of glucose concentration on NAT-induced insulin secretion as well as the influence of NAT on glucose-stimulated insulin secretion (GSIS). Such an interaction was also studied with the comparator insulinotropic agents like glyburide (GLY) and repaglinide (REP). Our results indicated that the islet secretory response to glucose stimulation was sensitized by NAT and REP, but not by the SUs, GLY and GLI. In addition, stimulation of insulin secretion *in vitro* by NAT was glucose-dependent while the effects of REP and GLY showed little or no glucose-sensitivity.

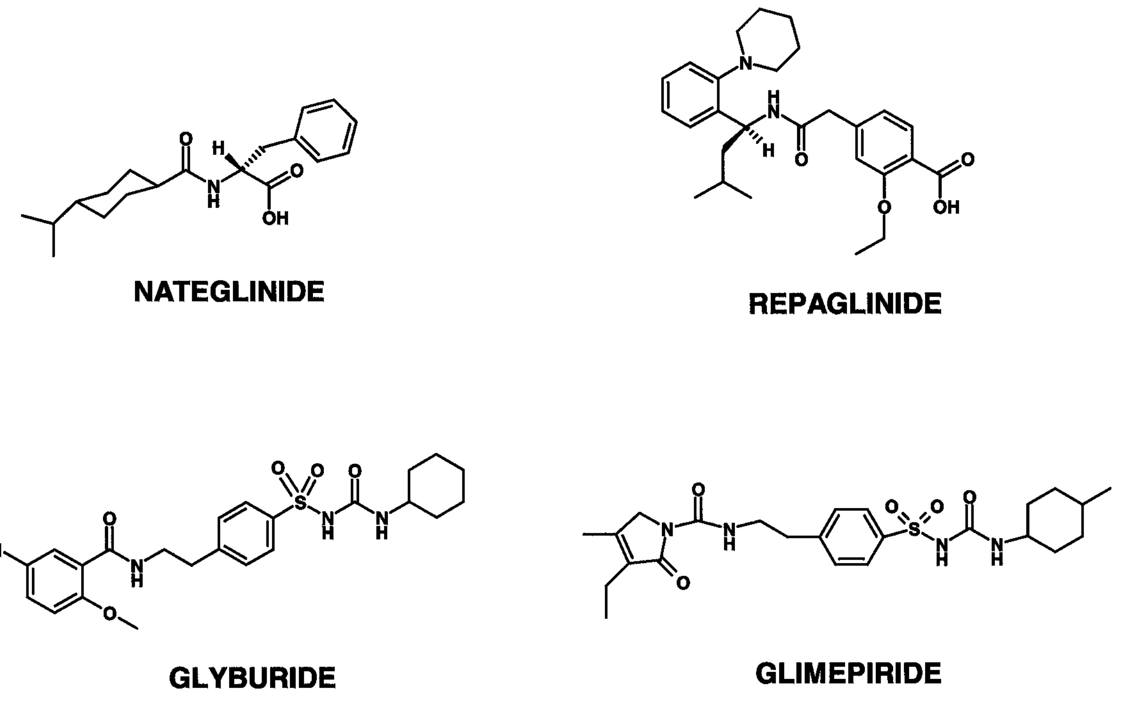

FIGURE 1 Chemical structure of hypoglycemic agents NAT, GLY, GLI, and REP.

### MATERIALS AND METHODS

### Islet Isolation

Pancreas were dissected from normal fed male Sprague Dawley rats (250–275 g), which were euthanized with Na pentobarbital i.p. at 120 mg/kg. Islets of Langerhans were isolated by librase digestion (0.5 mg/ml, Boebringer Mannheim, Germany) followed by a Ficoll gradient centrifugation.[16]

### Islet Static Incubation Assay

Freshly isolated islets were handpicked under a stereomicroscope by gentle suction through a large firepolished pipette (~400 μm diameter) into 60 × 15 mm Petri dishes (Corning) 25 ml of DMEM (Dulbecco's Modified Eagle Medium, Gibco BR) supplemented with 5 mM glucose (G5-DMEM) and 1% BSA (BSA was present in all incubation media throughout the experiments). Islets were preincubated in a humidified atmosphere of 95% $O_2$ and 5% $CO_2$ at 37°C for 1 hour. At the end of incubation, G5-DMEM medium was discarded and replaced with glucose free DMEM (G0-DMEM). Islets were then picked (2 islets/tube; 4 tubes/condition) into borosilicate glass tubes (12 × 75 mm) containing 500 μl chilled G0-DMEM. After islets were settled, 500 μl 2x treatment of the acute glucose/drug concentrations was added to each tube (1 ml final volume).

In the first study, the concentration-dependence of *in vitro* insulin secretion induced by hypoglycemic agents during 1 h static incubation in the presence of low (3 mM, G3), moderately elevated (8 mM, G8) and severely elevated (16 mM, G16) glucose was investigated. Each drug at 6–7 concentrations (4 tubes/concentration) including drug free control in the presence of G3, G8 and G16 were orderly set in a rack. Tubes were incubated at 37°C with intermittent hand shaking for 1 hour. In the second study, insulin secretion during one hour incubation at eight glucose concentrations (0, 3, 5, 6.5, 8, 9.5, 11 and 16 mM, 4 tubes/each glucose concentration) was measured in the presence or absence of one test drug (NAT, GLY, GLI or REP) at comparably effective concentrations approximately 2x respective $EC_{50}$s at G8. At the end of 1 h static incubation, islet media (500 μl/tube) were transferred to 96 deep-well plates and stored at −20°C for subsequent insulin analysis.

### Insulin Scintillation Proximity Assay (SPA)

The incubation media were diluted by factors ranging from lx to 20x depending on the concentrations of glucose/drugs/inhibitors. The diluted media were assayed for insulin content with SPA.[17] The assay employed commercially available products including a guinea pig anti-rat insulin specific antibody (Linco Research Inc) and scintillation proximity Type I reagent coupled to protein A (Amersham Life Science), and was performed as a single step assay. All samples were assayed in duplicate. The preparation of 96 well sample plates was made by sequentially pipetting standard/unknown samples, anti-insulin serum, $^{125}$I-insulin tracer, and SPA reagent, and the final volume equaled 175 μl/well. The plates were incubated and vortexed on a titer plate shaker for approximately 18–20 hours overnight at room temperature before being placed into a Wallac Microbeta 1450 Liquid Scintillation Counter to be read under a normalization protocol. The output was in counts per minute (CPM).

### Data Analysis

The sample insulin concentration was calculated by utilizing a template set up in Excel spreadsheet that possessed statistical analysis functions. The calculated concentration was eventually adjusted to reflect the degree of dilution. The intra- and inter-assay coefficients of variation were generally between 5% and 8%. $EC_{50}$s were calculated from 5-points dose-response curves fit with 4-parameter Hill sigmoidal equation in Sigmaplot (version 4.01).

However, in cases where high concentrations of glucose/drugs caused a decrease of insulin release, the values were excluded from the curve fitting analysis. Statistical significance was determined with t-test (single-tailed). $P < 0.05$ was considered significantly different.

### RESULTS

### GSIS in Nutrient-free and Nutrient-rich Incubation Media

To validate the islet static incubation assay and insulin SPA assay, we investigated GSIS in physiologically relevant nutrient-rich medium DMEM or in nutrient-free buffers such as Phosphate Buffered Saline (PBS) and Krebs Ringer Bicarbonate (KRB). While DMEM was rich in amino acids, the salines had glucose as the sole exogenous substrate. In all three types of media, insulin secretion from freshly isolated islets during 1 hour static incubation was stimulated by glucose in a concentration-dependent fashion, as shown in Figure 2. The characteristics of GSIS, however, differed considerably from medium to medium in several aspects: (1) the basal level of insulin release (at G0) was significantly lower in DMEM and KRB than in PBS; (2) the stimulation factor was 3.5-fold in PBS, 16.2-fold in KRB, and 26.7-fold in DMEM as glucose concentration increased from 0 to 16 mM; (3) the $EC_{50}$s (glucose concentration at which a half-maximal insulin release was achieved) were 5.8 mM and 6.0 mM, respectively, in PBS and DMEM; (4) in KRB, there was no significant increment in GSIS at glucose concentration up to 10 mM. Our results reinforce the importance of selection of incubation media and the presence of exogenous nutrients (*e.g.*, amino acids) for islet function, as have been repeatedly discussed by others.[18–20] Thus, DMEM seems to be an optimal choice for the study of insulin secretory response in isolated islets.

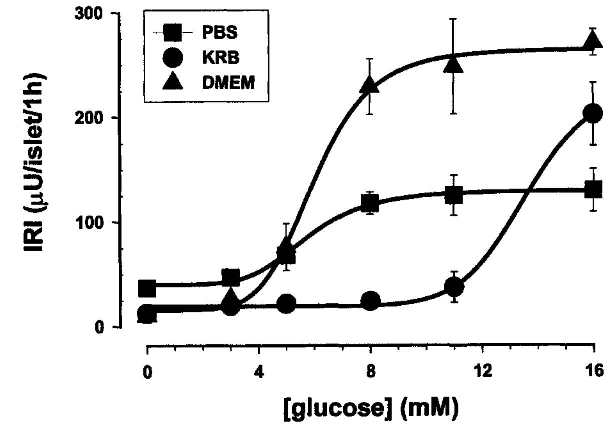

FIGURE 2 GSIS in various incubation media: DMEM (triangle), PBS (square), and KRB (circle). Data are the mean ± SEM (n = 4). In some cases the error bars are smaller than the dimension of the symbols and are therefore not visible. Ordinate shows the cumulative amount of insulin during 1 hour incubation in μU/islet and 1 μU equals 40 pg insulin; abscissa indicates glucose concentration in mM. Curves were fitted with 4-parameter sigmoidal Hill equation.

### Influence of Glucose on Secretagogue-Induced Insulin Secretion (Study 1)

The basal insulin secretion (in the absence of drug) during 1 h incubation was, respectively, 20.3 ± 2.4, 62.4 ± 2.3, and 192.6 ± 13.5 μU/islet at G3, G8, and at G16 (n = 96), demonstrating a glucose-dependence of insulin secretion. The ability of NAT, GLY and REP to stimulate insulin secretion was evaluated when the glucose concentration was maintained at 3 (G3), 8 (G8), or 16 (G16) mM. Representative results with REP at six concentrations are shown in Figure 3, in which the amount of insulin secretion at each glucose level in the absence (basal) and presence of REP are displayed. The basal insulin secretion in the absence of REP (shown with empty symbols) increased as glucose concentration is elevated. REP stimulated insulin secretion in a concentration-dependent manner at all glucose concentrations tested.

Parallel studies were carried out with NAT and GLY. The concentration-response curves for all drugs tested at G3, G8 and G16 were pooled and shown, respectively, in Figures 4A, B, and C. Drug-induced insulin secretion was expressed

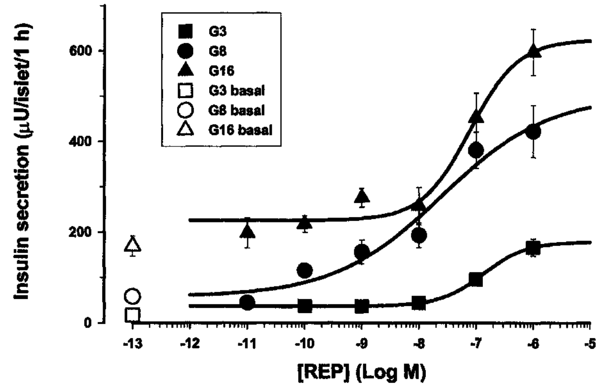

FIGURE 3 REP-stimulated insulin release from isolated rat islets during 1 hour static incubation at glucose concentrations of 3 mM (filled square), 8 mM (filled circle), and 16 mM (filled triangle). The corresponding empty symbols on the left side of the curves denote the respective basal insulin level in the absence of REP. Points represent data pooled from 4 independent experiments each with 4 tubes/condition (*i.e.*, n = 16/condition). Ordinate shows insulin secretion (μU/islet) during 1 hour incubation, and 1 μU equals 40 pg insulin; abscissa indicates REP concentration (M) in a logarithm scale.

as percent increase from basal (*i.e.*, stimulation factor) and $EC_{50}$ values were determined from the least square fitting of data with Hill sigmoidal equation and shown in Table I. The $EC_{50}$s with NAT was 14.2 μM at G3, decreased by 6-fold to 2.3 μM at G8 (NS), and by 16-fold to 0.9 μM at G16, suggesting sensitization of the insulinotropic effect of NAT by glucose. The potency of GLY remained statistically unchanged from G3 ($EC_{50}$ of 31.6 nM) to G8 ($EC_{50}$ of 41.2 nM), but decreased at G16 by 20-fold with an $EC_{50}$ of 0.6 μM. The $EC_{50}$ of REP had a moderate decrease of 5-fold at G8 (NS) compared to that at G3. There was, however, no further sensitization of REP effect at more elevated glucose (G16).

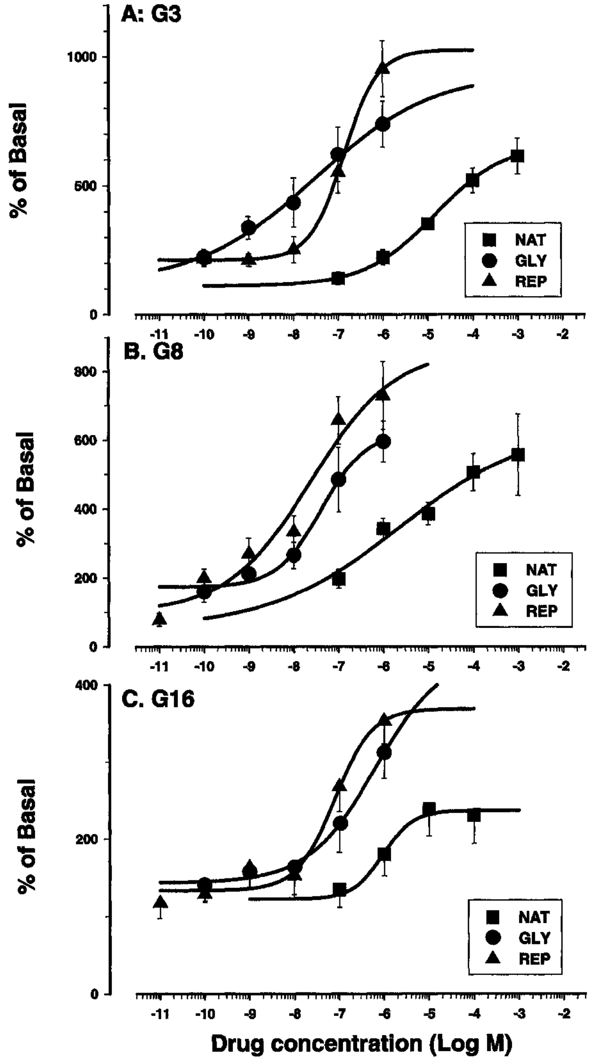

FIGURE 4 Concentration-dependent induction of insulin release from isolated rat islets during 1 hour static incubation by NAT (square), GLY (triangle) and REP (circle) in the presence of 3 mM, (Fig. 4A), 8 mM (Fig. 4B), and 16 mM glucose (Fig. 4C). Ordinate shows % increase in insulin release from the basal. Points represent data pooled from 4–6 independent experiments each with 4 tubes/condition (*i.e.*, n = 16–24/condition). Curves are 4-parameter Hill sigmoidal fitting of the points.

### Effect of NAT, GLY, GLI and REP on GSIS (Study 2)

Insulin secretion from rat isolated islets during 1 hour of static incubation at glucose concentrations of 1, 3, 5, 6.5, 8, 9.5, 11, and 16 mM were measured in the absence and presence of 5 μM NAT. This concentration of NAT was approximately 2x of the $EC_{50}$ of insulinotropic effect by NAT at glucose concentration of 8 mM (2.3 μM). Figure 5A illustrates the data of glucose-insulin response pooled from six independent experiments (n = 4 in each experiment). The

TABLE I $EC_{50}$s of insulinotropic effect of antidiabetic agents at three glucose levels

| Glucose | Nateglinide | Glyburide | Repaglinide |
|---|---|---|---|
| 3 mM | 14.2 μM | 31.6 nM | 0.1 μM |
| 8 mM | 2.3 μM | 41.2 nM | 24.7 nM |
| 16 mM | 0.9 μM* | 0.6 μM* | 78.5 nM |

* indicates significant difference compared to data in 3 mM glucose.

$EC_{50}$s were direct readouts of the parameters in curve fitting of the data in Figures 4A, B, and C with Hill 4-parameter sigmoidal equation using statistics function in Sigmaplot. In case where plateau has not been reached, the $EC_{50}$s were the values anticipated by the regression equation.

$EC_{50}$ values (glucose concentrations for a half-maximal GSIS) obtained directly from the parameters of Hill sigmoidal equation were $9.7 \pm 0.5$ mM in the absence of NAT and $8.0 \pm 0.5$ mM in the presence of NAT. The left shift of $EC_{50}$ in the presence of NAT may be partially interpreted as an increase in islet sensitivity to glucose. Moreover, the insulinotropic effect of NAT was additive to that of glucose, since the presence of NAT substantially increased the maximal value of GSIS.

Parallel studies on GSIS was performed in the absence and presence of hypoglycemic drugs GLY (100 nM), GLI (100 nM) and REP (50 nM). The concentrations of the drugs were so chosen that they were about equally effective in stimulating insulin secretion (approximately 2x respective $EC_{50}$s at G8 obtained from study 1). While GLI had not been tested in study 1, a

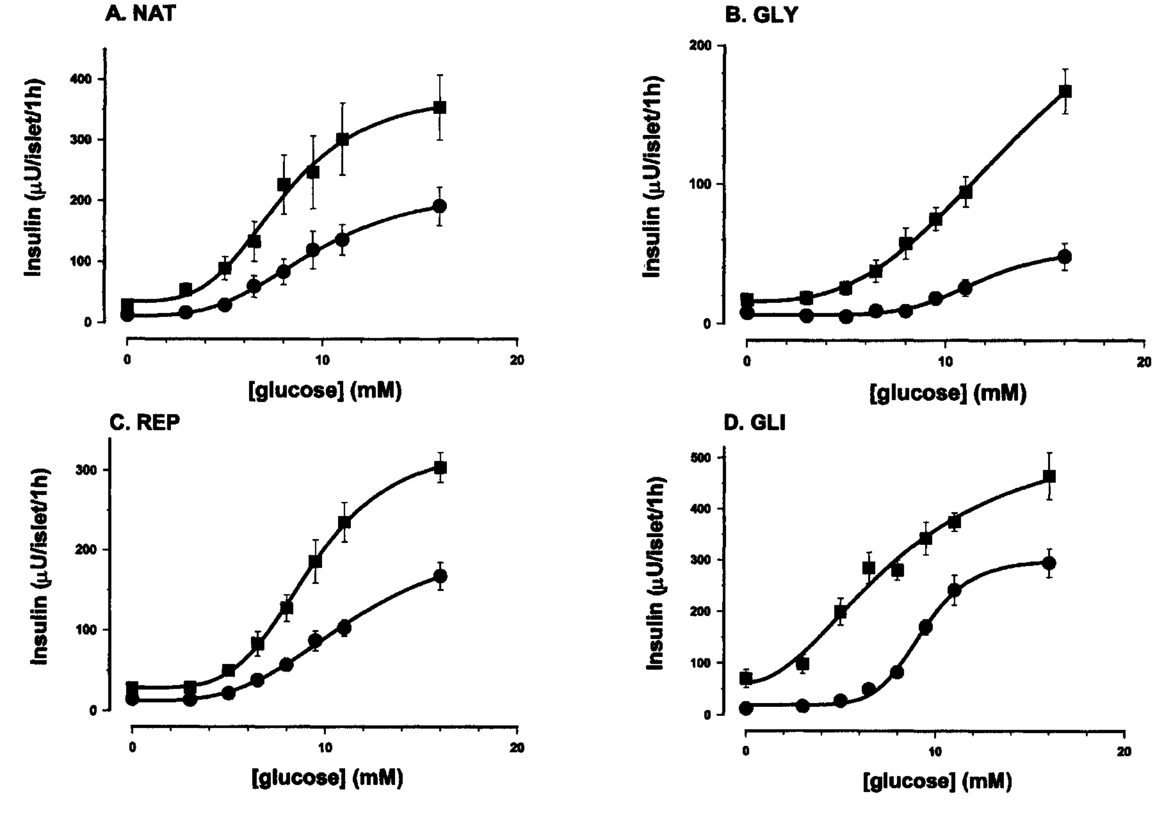

FIGURE 5 GSIS in the absence (circle) and presence (square) of 5 μM NAT (A), 100 nM GLY (B), 50 nM REP (C), and 100 nM GLI (D). Points represent data pooled from 4–6 independent experiments each with 4 groups/condition (*i.e.*, n = 16–24/condition). In some cases the error bars are smaller than the dimension of the symbols and are therefore not visible. Curves were fitted with 4-parameter sigmoidal Hill equation and $EC_{50}$s were directly read from fitting parameters. Ordinate shows the cumulative amount of released insulin during 1 hour incubation in μU/islet/1h (1 μU equals 40 pg of insulin); abscissa indicates glucose concentration in mM.

TABLE II Glucose concentration for a half-maximal GSIS (Mean ± SEM)

| Hypoglycemic drugs | Control (no drug) (mM) | 1 Drug (mM) |
|---|---|---|
| NAT | 9.7 ± 0.5 | 8.0 ± 0.5 * |
| GLY | 11.7 ± 0.7 | 15.1 ± 1.6* |
| GLI | 9.3 ± 0.1 | 8.4 ± 2.0 |
| REP | 11.6 ± 0.8 | 9.3 ± 0.1* |

Data are the average of 4–6 experiments, each with 4 groups/condition (*i.e.*, n = 16–24/condition).

concentration of 100 nM was used in this study, provided it was of similar potency with GLY. The concentration-response curves are shown, respectively, in Figures 5B, C and D. The $EC_{50}$s of GSIS with and without secretagogues are tabulated (Tab. II). The $EC_{50}$ of REP was reduced by 2.3 mM, a magnitude slightly greater than that with NAT, suggesting an increased sensitivity of islets to glucose. However, the SUs, GLY and GLI, caused, respectively, a pronounced increase (by 3.4 mM) and no change in $EC_{50}$s. In fact, GSIS in the presence of GLY hardly reached plateau at the highest glucose concentration tested (16 mM). The actual $EC_{50}$ might therefore be even greater than the value obtained from Hill sigmoidal fitting.

### DISCUSSION

The islet incubation assay in conjunction with insulin SPA assay adopted in this work allowed *in vitro* study of cumulative insulin secretion from pancreatic islets during static incubations. The method was validated through investigation of GSIS in nutrient-free or nutrient-rich incubation media. While in all media insulin was released in a glucose concentration-dependent manner, the composition of media profoundly altered the basic sigmoidal relationship. The lower insulin secretory capacity in nutrient-free salines may be attributable to multiple factors such as (1) impairment of glucose-sensing resulting from a reduced islet content of glucokinase; (2) decrease in rate of glycolysis resulting from insufficient activation of phosphofructokinase in response to a rise in hexose concentration; (3) reduction of insulin content in the islet cells.[21] It is therefore conceivable that nutrient-free salines are inappropriate for *in vitro* insulin study.

The present study assessed the influence of glucose on *in vitro* insulin secretion stimulated by hypoglycemic drugs. Our data showed that the augmentation by NAT of insulin release was glucose-sensitive, as evidenced by a respective 6- and 16-fold increase in potency with an elevation of glucose from 3 mM to 8 and 16 mM. These changes, albeit not drastic, indicated an ability of NAT to "self-correct" for the maintenance of glucose homeostasis. These data are in qualitative agreement with findings from studies utilizing the buffer-perfused pancreas[22] as well as from *in vivo* studies in rat or dog.[11,12] The glucose sensitization of the NAT's insulinotropic action *in vitro* and *in vivo* and the glucose desensitization of GLY's action are also consistent with the observations that NAT allows whereas SUs prevent nutrient-stimulated protein biosynthesis in β cells.[23,24]

The glucose-sensitive insulinotropic effect of NAT predicts that it would be a more effective drug in hyperglycemic patients than in normal individuals, depending, of course on the "health" of the pancreas or the residual *β*-cell mass. In addition, the reduced insulinogenic potency of NAT at low glucose level may be translated to an increased safety margin due to reduced propensity of serious hypoglycemia. Conversely, GLY showed a greater potency at low glucose concentrations, which may contribute to relatively high risk of hypoglycemia known to be associated with GLY therapy.[25–27] The insulinotropic effect of REP was more potent by 5-fold in the presence of moderately high glucose (8 mM) than in the presence of low glucose (3 mM). The sensitivity to glucose, however, diminished at high glucose level of 16 mM.

The result of enhanced effectiveness of NAT to stimulate insulin secretion at elevated than normal glucose level is consistent with the results from the study on $K_{ATP}$ channel,[15] which revealed a similar tendency of glucose-sensitivity, *i.e.*, NAT blocks $K_{ATP}$ channels more potently in high glucose (G16) than in normal glucose (G5). The $EC_{50}$s of NAT (14.2 μM) and GLY (31.6 nM) to stimulate insulin secretion at G3 correlate well with the $IC_{50}$s of NAT (7.4 μM) and GLY (16.6 nM) to block $K_{ATP}$ channel in β-cells in the presence of physiological glucose. However, the $EC_{50}$ of REP to stimulate insulin secretion in the study (134 nM) was significantly higher than would be expected based on $IC_{50}$ obtained from $K_{ATP}$ channel data (5 nM). The reason(s) for such a discrepancy are yet to be established.

We found that the insulinotropic drugs tested differentially altered the *in vitro* GSIS. NAT and REP appeared to sensitize the secretory response of islets to glucose in two ways: (1) their effect was additive to the effect of glucose, as evidenced by an increase in maximal insulin release in the presence of drugs (Figs. 5A and 5C); (2) both drugs left-shifted the $EC_{50}$s for GSIS, suggesting an increased islet sensitivity to glucose. The interpretation for such an interaction between drugs and glucose is lacking at present. Some recent studies indicated that GSIS cannot be attributed solely either to the closing of $K_{ATP}$ channels following an increase in intracellular ATP/ADP, or the role of glucose as a nutrient to cover the energy expenditure in the islet cells [28]. On the other hand, the insulinotropic action of NAT is likely to be mediated by $K_{ATP}$ channel-independent as well as $K_{ATP}$ channel-dependent pathways, while that of REP appears to be solely due to its closing of $K_{ATP}$ channels in β-cells.[7,29] Taken together, the closure of $K_{ATP}$ channels may not be sufficient to account fully for all the effects of hypoglycemic agents upon glucose sensitivity as well as other biophysical and biochemical variables in the islet cells.

The reported results on the effect of SUs on GSIS are rather controversial and lack consensus. The data in this study with SUs showed that the insulinotropic effect of GLY and GLI was additive to glucose stimulated insulin release (Figs. 5B and 5D), since the maximal insulin release was markedly increased in the presence of SUs. These agents, however, failed to increase sensitivity of islets to glucose as indicated by an increase (with GLY) or no change (GLI) of their $EC_{50}$s. The data are in agreement with the *in vivo* results of Groop *et al.*[30] and Ligtenberg *et al.*,[31] but deviate from those of Veneman *et al.*,[32] who reported that gliclazide, another SU, caused an apparent enhancement of β-cell glucose sensitivity.

The mechanism(s) by which the non-SU insulinotropic drugs (NAT and REP) and the SUs (GLY and GLI) exerted differential effect on GSIS are yet to be established. It is speculated that the non-SU drugs bind to the SU receptor at a molecular site distinct from that for SUs and hence interact with glucose in a distinct pattern. In this context, the existence of a common SU receptor with distinct sites for GLY and REP[8] and the presence of a specific binding site for NAT in addition to a common SU receptor[33] have been proposed to be responsible for the common and differential insulin-stimulating processes by these drugs. Alternatively, the sensitizing or desensitizing efficacy of hypoglycemic agents on GSIS may be linked to differences in their capacity to be inserted into the phospholipid domain of the plasma membrane. SUs, GLY and GLI, have been known to be internalized into β-cells to exert their action[21,34] while NAT appears to act extracellularly.[35] This distinction may lead to different modification of responsiveness to $Ca^{2+}$ of the effector system for insulin release and in turn, different glucose-sensitivity of islets.

In conclusion, nateglinide demonstrated a glucose-dependent and glucose-sensitizing insulinotropic action on isolated rat islets. These properties further distinguish nateglinide from other SU receptor ligands, raise the question of whether $K_{ATP}$-channel closure is the sole mechanism of action of this agent and predict a low hypoglycemic potential during therapeutic use of nateglinide.

### References

[1] Cook, D. L. and Hales, C. N. (1984). Intracellular ATP directly blocks $K^+$channels in pancreatic β-cells, *Nature*, **311**, 271-273.

[2] Orci, L. (1985). The insulin factory: a tour of the plant surroundings and a visit to the assembly line, *Diabetologia*, **28**, 528-546.

[3] Sturgess, N. C., Ashford, M. L., Cook, D. L. and Hales, C. N. (1985). The sulphonylurea receptor may be an ATP-sensitive potassium channel, Lancet, **2**(8453), 474-475.

[4] Dunne, M. J., Ilott, M. C. and Petersen, O. H. (1987). Interaction of diazoxide, tolbutamide and ATP on nucleotide-dependent $K^+$ channels in an insulin-secreting cell line, *J. Membr. Biol.*, **99**(3), 215-224.

[5] Schwanstecher, M., Manner, K. and Panten, U. (1994). Inhibition of $K^+$ channels and stimulation of insulin secretion by the sulfonylurea, glimepiride, in relation to its membrane binding in pancreatic islets, *Pharmacology*, **49**, 105-111.

[6] Gromada, J., Dissing, S., Kofod, H. and Frokjaer-Jensen, J. (1995). Effects of the hypoglycemic drugs repaglinide and glibenclamide on ATP-sensitive potassium-channels and cytosolic calcium levels in β TC3 cells and rat pancreatic beta cells, *Diabetologia*, **38**, 1025-1032.

[7] Malaisse, W. J. (1995). Stimulation of insulin release by non-sulfonylurea hypoglycemic agents: the meglitiide family, *Horm. Metab. Res.*, **27**, 263-266.

[8] Fuhlendorff, J., Rorsman, P., Kofod, H., Brand, C. L., Rolin, B., MacKay, P., Shyniko, and R. Carr, R. D. (1998). Stimulation of insulin release by repaglinide and glibenclamide involves both common and distinct processes, *Diabetes*, **47**, 345-351.

[9] Akiyoshi, M., Kakei, M., Nakazaki, M., Tanaka, H. (1995). A new hypoglycemic agent, A-4166, inhibits ATP-sensitive potassium channels in rat pancreatic β-cells, *Am. J. Physiol.*, **268**, E185-193.

[10] Tsukuda, K., Sakurada, M., Niki, I., Oka, Y. and Kikuchi, M. (1998). Insulin secretion from isolated rat islets induced by the novel hypoglycemic agent A-4 166, a derivative of D-phenylalanine, *Horm. Metab. Res.* **30**, 42-49.

[11] Mark, M., Grell, W. (1997). Hypoglycemic effects of the novel antidiabetic agent repaglinide in rat and dogs, *Br. J. Pharmacol.* **121**, 1597-1604.

[12] De Souza, C. J., Russo, P., Lozito, R. and Dunning, B. (1997). The metabolic advantages of a rapid onset/ short acting insulin secretagogue on prandial glucose excursions, *Diabetes*, **46**, 241A.

[13] Seto, Y., Fujita, H., Dan, K., Fujita, T. and Kato, R. (1995). Stimulating activity of A-4166 on insulin release in *in situ* hamster pancreatic perfusion, *Pharmacology*, **51**, 245-253.

[14] Ikenoue, T., Akiyoshi, M., Fujitani, S., Okazaki, K., Kondo, N. and Maki, T. (1997). Hypoglycemic and insulinotropic effects of a novel oral antidiabetic agent, (−)-N-(trans-4-isopropylcyclohexane-carbonyl)-D-Phenylalanine (A-4 166), *Br. J. Pharmacol.* **120**, 137-145.

[15] Hu, S. and Wang, 5. (1998). Effect of antidiabetic agent, netaglinide, on $K_{ATP}$ channel in β-cells:comparison to glyburide and repaglinide, *Diabetologia*, **41 Suppl**, A139.

[16] Lacy, P. E. and Kostianovsky, M. (1967). Method for the isolation of intact islets of Langerhans from the rat pancreas, *Diabetes*, **16**, 35-39.

[17] Cook, N. D. (1996). Scintillation proximity assay: a versatile high-throughput screening technology, *Drug. Disc. Trends*, **1**, 287-294.

[18] Malaisse, W. J., Lea, M. A. and Malaisse-Lagae, F. (1968). The effect of mannoheptulose on the phosphorylation of glucose and the secretion of insulin by islets of Langerhans, *Metabolism*, **17(2)**, 126-132.

[19] Zawalich, W. S., Pagliara, A. S. and Matschinsky, F. M. (1977). Effects of iodoacetate, mannoheptulose and 3-O-methyl glucose on the secretory function and metabolism of isolated pancreatic islets, *Endocrinology*, **100**, 1276-1283.

[20] Escolar, J. C., Hoo-Paris, R., Castex, C. and Sutter, B. C. (1990). Effect of low temperatures on glucose-induced insulin secretion and glucose metabolism in isolated pancreatic islets of the rat, *J. Endocrinol.*, **125**, 45-51.

[21] Malaisse, W. J. (1992). Insulin biosynthesis and secretion *in vitro*. Chapter II, In: *International textbook of diabetes mellitus*. (Alberti, K. G. M. M., Defronzo, R. A., Keen, H. and Zimmet, P. Ed.). John Wiley & Sons, pp. 261-283.

[22] Morimoto, S., Mokuda, O. and Sakamoto, Y. (1998). Ay-4166 increases the sensitivity of insulin secretion to glucose in isolated perfused rat pancreas, *Horm. Metab. Res.*, **30**, 77-79.

[23] Levy, J. and Malaisse, W. J. (1975). The stimulus-secretion coupling of glucose-induced insulin release-XVII. Effects of sulfonylureas and diazoxide on insular biosynthetic activity. *Biochemical Pharmacology*, **24**(2), 235-239.

[24] Vinambres, C., Villanueva-Penacarrillo, M. L., Valverde, I. and Malaisse, W. J. (1996). Repaglinide preserves nutrient-stimulated biosynthetic activity in rat pancreatic islets, Pharmacological Research, **34**(1-2), 83-85.

[25] Asplund, K., Wiholm, B. E. and Lithner, F. (1983). Glibenclamide-associated hypoglycemia: a report on 57 cases, *Diabetologia*, **24**, 412-417.

[26] Ferner, R. E. and Neil, H. A. W. (1988). SUs and hypoglycemia, *Br. Med. J.*, **296**, 949-950.

[27] Seltzer, H. S. (1989). Drug-induced hypoglycemia, Endocrinol. *Metab. Clin. North. Am.*, **18**, 163-183.

[28] Malaisse, W. J. (1997). Stimulation of insulin release by D-glucose in depolarized pancreatic islets, *Cell Signal*, **9**, 265-268.

[29] Fujitani, S., Ikenoue, T., Akiyoshi, M., Maki, T. and Yada, T. (1996). Somatostatin and insulin secretion due to common mechanisms by a new hypoglycemic agent, A-4166, in perfused rat pancreas, *Metabolism*, **45**, 184-189.

[30] Groop, L. C., Ratheiser, K., Luzi, L., Melander, A., Simonson, D. C., Petrides, A., Bonadonna, R. C., Widen, E. and DeFrotizo, R. A. (1991). Effect of sulphonylurea on glucose-stimulated insulin secretion in healthy and non-insulin dependent diabetic subjects: a dose-response study, *Acta Diabetol.*, **28**, 162-168.

[31] Ligtenberg, J. J., Venker, C. E., Sluiter, W. J., Reitsma, W. D. and Van Haeften, T. W. (1997). Effect of glibenclamide on insulin release at moderate and high blood glucose levels in normal man, *Eur. J. Clin. Invest.*, **27**(6), 685-689.

[32] Veneman, T. F., van Haeften, T. W. and Veen, E. A. (1991). Effect of acute administration of gliclazide on the glucose sensitivity of pancreatic B-cells in healthy subjects, *Clin. Sci.*, **81**, 101–106.
[33] Fujita, T., Seto, Y., Kondo, N. and Kato, R. (1996). Studies on the N-[(trans-4-isopropylcyclohexyl)-carbonyl]-D-phenylalanine (A-4166) receptor in HIT T-15 cells, *Biochem. Pharmacol.*, **52**, 407–411.
[34] Marynissen, G., Smets, G., Loppel, G., Gerlache, L. and Malaisse, W. J. (1992). Internalization of glimepiride in the pancreatic B-cell, *Acta Diabetol.*, **29**, 113–114.
[35] Malaisse-Lagae, F. and Malaisse, W. J. (1996). Fate of 3H- and 14C-labelled A-4166 in pancreatic islets, *Acta Diabetologica*, **33(4)**, 298–300.

